# Alexithymia Is Associated with Greater Risk of Chronic Pain and Negative Affect and with Lower Life Satisfaction in a General Population: The Hisayama Study

**DOI:** 10.1371/journal.pone.0090984

**Published:** 2014-03-12

**Authors:** Mao Shibata, Toshiharu Ninomiya, Mark P. Jensen, Kozo Anno, Koji Yonemoto, Seiko Makino, Rie Iwaki, Koji Yamashiro, Toshiyuki Yoshida, Yuko Imada, Chiharu Kubo, Yutaka Kiyohara, Nobuyuki Sudo, Masako Hosoi

**Affiliations:** 1 Department of Psychosomatic Medicine, Graduate School of Medical Sciences, Kyushu University, Fukuoka, Japan; 2 Department of Psychosomatic Medicine, Kyushu University Hospital, Fukuoka, Japan; 3 Department of Environmental Medicine, Graduate School of Medical Sciences, Kyushu University, Fukuoka, Japan; 4 Department of Medicine and Clinical Science, Graduate School of Medical Sciences, Kyushu University, Fukuoka, Japan; 5 Department of Rehabilitation Medicine, University of Washington, Seattle, Washington, United States of America; 6 Biostatistics Center, Kurume University, Fukuoka, Japan; 7 Department of Speech and Hearing Sciences, International University of Health and Welfare, School of Health Sciences at Fukuoka, Fukuoka, Japan; Hokkaido University, Japan

## Abstract

**Introduction:**

Chronic pain is a significant health problem worldwide, with a prevalence in the general population of approximately 40%. Alexithymia — the personality trait of having difficulties with emotional awareness and self-regulation — has been reported to contribute to an increased risk of several chronic diseases and health conditions, and limited research indicates a potential role for alexithymia in the development and maintenance of chronic pain. However, no study has yet examined the associations between alexithymia and chronic pain in the general population.

**Methods:**

We administered measures assessing alexithymia, pain, disability, anxiety, depression, and life satisfaction to 927 adults in Hisayama, Japan. We classified the participants into four groups (low-normal alexithymia, middle-normal alexithymia, high-normal alexithymia, and alexithymic) based on their responses to the alexithymia measure. We calculated the risk estimates for the criterion measures by a logistic regression analysis.

**Results:**

Controlling for demographic variables, the odds ratio (OR) for having chronic pain was significantly higher in the high-normal (OR: 1.49, 95% CI: 1.07–2.09) and alexithymic groups (OR: 2.56, 95% CI: 1.47–4.45) compared to the low-normal group. Approximately 40% of the participants belonged to these two high-risk groups. In the subanalyses of the 439 participants with chronic pain, the levels of pain intensity, disability, depression, and anxiety were significantly increased and the degree of life satisfaction was decreased with elevating alexithymia categories.

**Conclusions:**

The findings demonstrate that, in the general population, higher levels of alexithymia are associated with a higher risk of having chronic pain. The early identification and treatment of alexithymia and negative affect may be beneficial in preventing chronic pain and reducing the clinical and economic burdens of chronic pain. Further research is needed to determine if this association is due to a causal effect of alexithymia on the prevalence and severity of chronic pain.

## Introduction

Chronic pain is a common problem, with prevalence estimates at approximately 40% of the general population [1], [2]. The impact of pain on economies is enormous. For example, the cost of back pain alone is equivalent to more than one-fifth of one country's total health expenditure and to 1.5% of the annual gross domestic product of the UK [3]. In addition to its economic impact, chronic pain is arguably one of the health care issues with the greatest negative impact on quality of life.

Chronic pain is known to have significant biological, psychological, and social causes and consequences [4]–[6], and thus adequate pain assessments and treatments should address all of these factors. In order to expand the potential targets of pain treatment and therefore help minimize the prevalence of the negative impacts of chronic pain, research is needed to identify the biopsychosocial factors that are most consistently associated with pain and pain-related outcomes.

A potential factor that may contribute to the development and maintenance of chronic pain is alexithymia [7], [8]. Alexithymia is the label used to describe a personality trait associated with an inability to regulate negative affect [9]. The term is derived from Greek, and literally means “a lack of words for feelings” [10]. Alexithymia is a disturbance of both cognitive and affective functioning characterized by difficulty in recognizing or describing one's emotions. The most common measure of alexithymia is the 20-item Toronto Alexithymia Scale (TAS-20) [11]. The TAS-20 assesses three components of alexithymia: (1) difficulty identifying feelings (DIF); (2) difficulty describing feelings (DDF); and (3) externally-oriented thinking (EOT).

In our previous research, we found a measure of alexithymia to be positively associated with pain intensity and interference, and negatively associated with vitality in a sample of individuals with neuromuscular disease and chronic pain [12]. We also found evidence that the effects of alexithymia on pain may be mediated by negative affect [13]. Additionally, research in pain populations by our group and others has identified the TAS-20 DIF scale as the most consistent factor associated with chronic pain and pain-related dysfunction [7], [14], [15]. However, the studies addressing the relationship between alexithymia and chronic pain to date have used participants who are not necessarily representative of the population (e.g., patients, transit workers, and students), which limits the generalizability of extant findings. To our knowledge, there are no studies examining the role that alexithymia might play in comprehensive chronic pain in a general population.

To elucidate this association, we performed a population-based cross-sectional survey in a Japanese community. We hypothesized that (1) the measure of alexithymia is associated with chronic pain, (2) this association is mediated by negative affect, and (3) the DIF domain of alexithymia is associated with criterion measures stronger than the other components of alexithymia. Our planned analyses regarding the associations between alexithymia and measures of additional pain-related outcomes (specifically, pain intensity, depression, anxiety, disability, and life satisfaction) were considered exploratory, as these associations have not yet been tested in prior research.

## Methods

### 1. Study participants

The Hisayama Study is an ongoing, long-term cohort study examining cardiovascular disease and its risk factors in Hisayama, a suburban town adjoining Fukuoka City, a metropolitan area in southern Japan. Full community surveys of the health status of residents aged 40 and older have been repeated every five years since 1961 [16]. Data for the present study were taken from responses to questions regarding pain and psychological functioning included in the whole survey administered in 2010.

Among 2,223 residents aged 40 and older who participated in the health survey, a total of 1,027 residents (participation rate: 46%) consented to participate in these questions of the 2010 whole survey. Of these, 66 had missing data and 34 did not complete the questionnaires, leaving a final sample of 927 participants (326 men and 601 women) (Fig. S1). This study was approved by the Kyushu University Institutional Review Board for Clinical Research. Written informed consent was obtained from all participants.

### 2. Measures

#### 2.1. Alexithymia

Alexithymia was assessed for each participant using the 20-item Toronto Alexithymia Scale (TAS-20), which is the most psychometrically valid measurement of alexithymia [11]. As mentioned above, the TAS-20 consists of 20 statements that reflect three domains of alexithymia: (1) DIF; (2) DDF; and (3) EOT. Each item is rated on a 5-point Likert scale, with 1 = “strongly disagree” and 5 = “strongly agree.”

We classified the participants as alexithymic (TAS-20 score >60) or non-alexithymic (TAS-20 score ≤60) based on their total TAS-20 scores according to previous studies [9], and subsequently classified the non-alexithymic group into three subgroups: low-normal alexithymia (score <44), middle-normal alexithymia (score 44–50) and high-normal alexithymia (score 51–60) based on their tertile values. In addition to the tertile values in this study, a total score of 51–60 has been defined as “borderline alexithymia” or “possible alexithymia” in some studies [17]. We also divided the TAS-20 subscale scores of the three domains into quartiles, as there are no published cutoffs for classifying individuals into alexithymic groups for the TAS-20 subscales. The Japanese version of the TAS-20 has been shown to be both reliable and valid [18].

#### 2.2. Assessment of presence of acute and chronic pain

The International Association for the Study of Pain (IASP) defines pain as “an unpleasant sensory and emotional experience associated with actual or potential tissue damage, or described in terms of such damage” and also explains that “Pain is always subjective.” The definition of ‘chronic’ pain based on duration has not been clearly established, but 3 or 6 months or more is generally used [19], [20]. According to this definition, we defined chronic pain as having any subjective pain for more than 6 months.

As part of the Hisayama Study health survey, the participants were asked to indicate whether they experienced any pain and how long the pain has lasted. Those who reported <6 months of pain and those with pain that had been experienced for 6 months or longer were classified as having acute (i.e., recent onset) and chronic pain, respectively. For complementary information, the participants with pain were asked to select their primary pain site using the IASP site categories [19]: 1 = head and face, 2 = neck, 3 = shoulder and arms, 4 = chest, 5 = back, 6 = stomach, 7 = low back, 8 = legs, 9 = pelvic and genital area, 10 = pain at more than one site.

#### 2.3. Pain intensity

The participants were asked to rate the average intensity of their pain in the past week on a 100-mm visual analogue scale (VAS). Anchors were “No pain” (0 mm) and “Pain as bad as it could be (100 mm).” A great deal of evidence supports the reliability and validity of the VAS as a measure of pain intensity [21].

#### 2.4. Disability

Participants were asked to rate their average disability in the past week on a 100-mm VAS. Anchors were “No disability” (0 mm) and “Disability as bad as it could be (100 mm) [22].”

#### 2.5. Life satisfaction

Participants were asked to rate their global life satisfaction on a 100-mm VAS. Anchors were “Feeling no life satisfaction at all” (0 mm) and “Feeling life satisfaction as good as it could be (100 mm) [23].”

#### 2.6. Negative affect

Negative affect was measured with the depression and anxiety scales of a Japanese version of the Symptom Checklist 90-revised (SCL-90-R). SCL-90-R scales have established validity and reliability [24].

#### 2.7. Demographic/descriptive variables and covariates

Age, sex, marital status, educational level, and economic status are factors that could potentially influence both pain and alexithymia and were therefore assessed and controlled in all analyses. Marital status was classified as never married, divorced, separated, widowed, married, or cohabiting. Educational level was classified as one of three education duration categories: under 9 years, 9–12 years, or over 12 years. Economic status was assessed by a question asking, ‘How difficult or easy is your current financial status?’ Response options for this question were ‘Very hard,’ ‘Hard,’ ‘Normal,’ ‘Easy,’ and ‘Very easy.’ Based on the participant's response, economic status was divided into three classes: low (very hard or hard), average (normal), and high (easy and very easy). Similar one-item questions about economic status have demonstrated validity through their associations with psychological and physical health [25].

### 3. Statistical analysis

We first computed the means and standard deviations, medians and interquartile ranges (of continuous variables), and rates (of categorical variables) of the study variables for descriptive purposes. To better understand the association between alexithymia and potential confounding factors (i.e., age, sex, marital status, educational level, and economic status), we examined their trend tests using a linear regression analysis, logistic regression analysis, or the Jonckheere-Terpstra test, as appropriate. Logistic regression analysis was used to examine the unadjusted and adjusted odds ratios (ORs) with 95% confidence intervals (CIs) and p for trend of chronic pain according to the TAS-20 score levels taken as categorical variables. In the multivariable-adjusted model, adjustments were made for age, sex, marital status, years of education, and economic status. We estimated the ORs per 1-point increment in the TAS-20 score using the relevant logistic model including the TAS-20 score taken as a continuous variable. The heterogeneity in the association between sexes was tested by adding the interaction term to the relevant logistic model. We also estimated the association (ORs and p for trend) between the quartiles of the TAS-20 subscales and the presence of chronic pain using logistic regression analysis. The trends in the dose-response associations between TAS-20 score levels and the pain intensity, disability, anxiety, depression, and life satisfaction were tested using the Jonckheere-Terpstra trend test for the participants with chronic pain. The SAS software package version 9.2 (SAS Institute, Cary, NC, USA) was used for all analyses. Two-sided values of p<0.05 were considered significant in all analyses.

## Results

The characteristics of the study sample are summarized in Table 1 as a function of the TAS-20 categories. The prevalence of alexithymic (TAS-20>60) in all participants was 7.8% (n = 72; 35 men, 37 women). There were significant associations between the TAS-20 categories and both education level and economic status, with lower education level and lower economic status associated with higher levels of the TAS-20 categories relative to those with higher education level or economic status. The scores for negative affect, such as depression and anxiety symptoms, were significantly increased with elevating TAS-20 categories. No significant associations were found between the TAS-20 categories and age, sex, or marital status.

**Table 1:** Characteristics of the study population according to the TAS-20 score level.

	Total	TAS-20 score				
		Low-normal	Middle-normal	High-normal	Alexithymic	
		<44	44–50	51–60	>60	p for trend
	n = 927	n = 278	n = 283	n = 294	n = 72	
***Sociodemographic characteristics***						
Age, year	61±11	60±11	61±11	61±11	63±13	0.10
Women, %	64.8	64.7	66.4	66.7	51.4	0.32
Marital status (married/cohabiting), %	81.8	86.0	78.1	80.6	84.7	0.33
Educational levels (under 9 years), %	17.0	11.2	15.9	21.4	26.4	<0.001
Economic status (low), %	19.6	17.3	16.3	22.8	29.2	0.01
***Negative affect***						
Depression symptom, score	0.69 (0.46–1.00)	0.54 (0.31–0.77)	0.62 (0.38–0.85)	0.85 (0.62–1.23)	1.31 (0.85–1.77)	<0.001
Anxiety symptom, score	0.40 (0.20–0.70)	0.20 (0.10–0.40)	0.30 (0.20–0.50)	0.60 (0.30–0.80)	1.00 (0.60–1.30)	<0.001

Values are means ± std. dev. or frequencies or median (interquartile range).

The TAS-20: the 20-item Toronto Alexithymia Scale.

Approximately 47% of the participants (n = 439; 152 men, 287 women) were classified as having chronic pain, and 17.9% (n = 166; 41 men, 125 women) were classified as having acute pain. For those with pain, the primary pain sites were the low back (30.1%), shoulders and arms (30.1%), legs (19.6%), head, face, or neck (13.2%), and other sites (7.2%) in the chronic pain group, and shoulders and arms (34.9%), low back (22.9%), legs (16.9%), head, face, or neck (15.0%), back (6.0%), and other sites (4.2%) in the acute pain group. The prevalence of alexithymic turned out to be 4.0% in the painless group, 6.6% in the acute pain group, and 10.9% in the chronic pain group.

**Figure 1:**
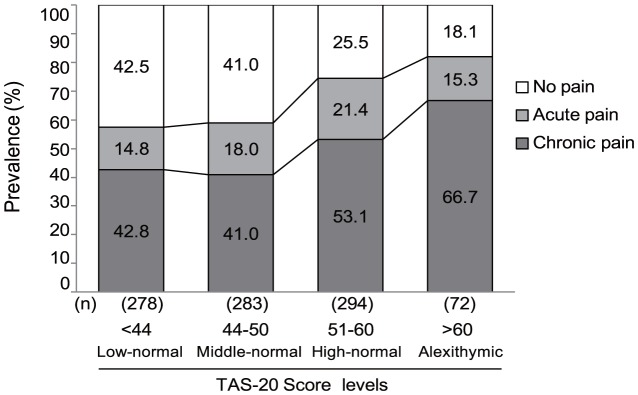
Self-reported pain prevalence according to the TAS-20 score levels in a general population from the Hisayama Study health survey. Acute pain: <6 months of pain. Chronic pain: pain that had been experienced for 6 months or longer.

The prevalence of pain as a function of the TAS-20 categories is shown in Figure 1. As the scores of the TAS-20 categories increased, the prevalence of chronic pain increased and that of ‘no pain’ decreased.

Table 2 shows the unadjusted and multivariable-adjusted ORs for the presence of chronic pain according to the TAS-20 categories. Compared with the low-normal alexithymia group, the unadjusted ORs for the presence of chronic pain were significantly higher, around twofold higher, in the high-normal alexithymia and alexithymic groups. After adjusting for age, sex, marital status, years of education, and economic status, this association remained substantially unchanged. Approximately 40% of the participants belonged to these two (high-normal alexithymia and alexithymic) high-risk groups.

**Table 2:** Odds ratios for chronic pain according to the TAS-20 category score.

	TAS-20 score	Number of participants	Number with Chronic pain	Unadjusted			Multivariable-adjusted		
Alexithymia level				OR (95%CI)	p value	p for trend	OR (95%CI)	p value	p for trend
***Total***									
Low-normal	<44	278	119	1.00 (reference)			1.00 (reference)		
Middle-normal	44–50	283	116	0.93 (0.66–1.30)	0.66	<0.001	0.91 (0.65–1.28)	0.6	<0.001
High-normal	51–60	294	156	1.51 (1.09–2.10)	0.01		1.49 (1.07–2.09)	0.02	
Alexithymic	>60	72	48	2.67 (1.55–4.61)	<0.001		2.56 (1.47–4.45)	0.001	
***Men***									
Low-normal	<44	98	40	1.00 (reference)			1.00 (reference)		
Middle-normal	44–50	95	38	0.97 (0.54–1.72)	0.91	0.007	0.96 (0.54–1.72)	0.9	0.01
High-normal	51–60	98	51	1.57 (0.89–2.77)	0.12		1.56 (0.88–2.77)	0.13	
Alexithymic	>60	35	23	2.78 (1.24–6.22)	0.01		2.55 (1.12–5.82)	0.03	
***Women***									
Low-normal	<44	180	79	1.00 (reference)			1.00 (reference)		
Middle-normal	44–50	188	78	0.91 (0.60–1.37)	0.64	0.004	0.89 (0.58–1.35)	0.58	0.005
High-normal	51–60	196	105	1.48 (0.97–2.22)	0.06		1.48 (0.98–2.25)	0.06	
Alexithymic	>60	37	25	2.66 (1.26–5.63)	0.01		2.59 (1.21–5.53)	0.01	

OR: odds ratio; CI: confidence interval.

Multivariable adjustment was made for age, gender, marital status, years of education and economic status. In the stratified analyses of gender, ORs were not adjusted for gender.

As a continuous variable, every 1-point increment in the TAS-20 score was associated with a 1.04-times (95%CI: 1.02–1.06) higher likelihood of the presence of chronic pain after the adjustment for the aforementioned confounding factors. The subgroup analysis stratified by sex showed that the odds ratios in the high-normal alexithymia and alexithymic groups were significant for both sexes, without any evidence of significant heterogeneity in the association between sexes (p for heterogeneity = 0.54).

Because including the participants with acute pain in the group without chronic pain in the present analysis may have underestimated the association, we examined the difference between the chronic pain and no-pain groups only. In these analyses, the odds ratios were even higher in the high-normal alexithymia group (OR: 2.05, 95% CI: 1.40–3.00) and alexithymic group (OR: 3.60, 95% CI: 1.83–7.08). The relationships between TAS-20 categories and chronic pain became nonsignificant after adjusting for depression symptoms (p for trend = 0.57) or for anxiety symptoms (p for trend = 0.57).

**Figure 2:**
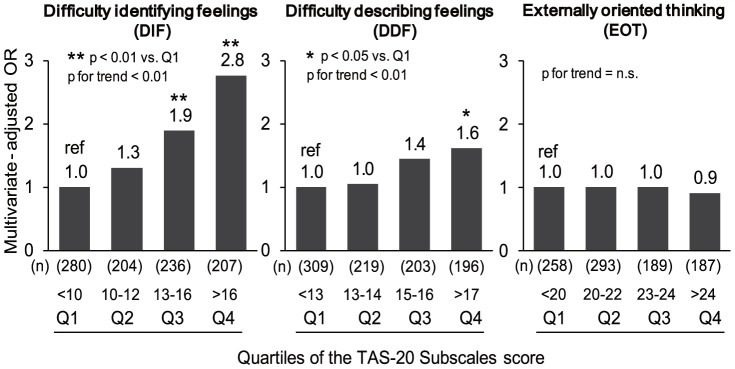
Odds ratios for chronic pain according to the TAS-20 subscales, adjusted for demographic factors in the general population.

Figure 2 presents the odds ratios for chronic pain as a function of quartiles of the TAS-20 subscales score, controlled for demographic factors. There were significant differences between the first quartile and both the third and the fourth quartiles in DIF subscale score. There was also a significant difference between the first quartile and the fourth quartile in DDF subscale score. However, there was no significant association between the EOT subscale score quartiles and the presence of chronic pain. Thus, the TAS-20 DIF subscale demonstrated the strongest association with the presence of chronic pain, although in this sample the TAS-20 DDF subscale may also play a role.

Table 3 shows the association between the TAS-20 categories and pain-related outcomes of the 439 participants with chronic pain. As the level of the TAS-20 categories increased, so did the levels of pain intensity, disability, and depression and anxiety symptoms. The TAS-20 categories were negatively associated with life satisfaction.

**Table 3:** The relationship between the TAS-20 score levels and the pain-related outcomes of the 439 participants with chronic pain.

	TAS-20 score				
	Low-normal	Middle-normal	High-normal	Alexithymic	
	<44	44–50	51–60	>60	p for trend
	n = 119	n = 116	n = 156	n = 48	
Pain intensity, mm	30 (15–50)	44 (20–54)	47 (24–65)	58 (36–80)	<0.001
Disability, mm	5 (0–15)	15 (0–31)	10 (0–38)	29 (3–61)	<0.001
Depression, score	0.7 (0.4–0.8)	0.7 (0.5–1.0)	0.9 (0.6–1.3)	1.4 (1.2–1.9)	<0.001
Anxiety, score	0.3 (0.2–0.6)	0.4 (0.2–0.6)	0.6 (0.4–0.8)	1.1 (0.8–1.6)	<0.001
Life satisfaction, mm	75 (50–89)	65 (47–81)	51 (40–71)	50 (38–61)	<0.001

Values are medians (interquartile range).

Pain intensity, disability and life satisfaction were evaluated by Visual Analogue Scale.

Depression and anxiety scores were evaluated by Symptom Check List-90-R.

## Discussion

To our knowledge, this is the first study to examine the association between alexithymia and chronic pain in a sample that is representative of the general population. As hypothesized, we found that alexithymia is significantly associated with a higher prevalence of chronic pain and that this association is mediated by negative affect, such as depression and anxiety symptoms. Also as hypothesized, the TAS-20 subscale assessing difficulty identifying feelings is more closely associated with pain than the other two TAS-20 subscales. We also found that alexithymia was associated with measures of additional pain-related quality of life domains (depression, anxiety, disability, and satisfaction with life) in the subsample of individuals with chronic pain. These findings have important implications for understanding pain and promoting general health.

### 1. Comparison with previous reports

The prevalence of alexithymic was reported to be approximately 10% in studies based in Finland (age range: 30–97 years) and Germany (age range: 20–69 years) [26], [27]. Our prevalence result, 7.8% (age range: 40–91 years), is probably lower because our participants did not include younger people (in their 20 s), who have been reported to have relatively high TAS-20 scores in Japan [18]. Although as far as we know there are no population-based studies on the relationship between alexithymia and chronic pain, there are some hospital-based cross-sectional studies for various patient populations. Most of these studies found a positive association between alexithymia and the presence of chronic pain [15], [28]–[30].

For example, Mehling and Krause reported that scoring in the upper quartile of the alexithymia total score was associated with twofold (adjusted OR = 2.00, 95%CI: 1.31–3.00) higher odds of the 12-month prevalence of low back pain, which was assessed by the medical history taken during the drivers' relicensing exams of 1,180 San Francisco transit operators [8]. These results are consistent with our finding. However, several studies have shown negative [31]–[33] or mixed correlations [34]. The discrepancy in correlations may be due to differences in health status or study design (e.g., using healthy controls or patient controls).

A population-based prospective longitudinal study [35] was conducted with the same population as that in the aforementioned cross-sectional study by Mehling and Krause [8]. The longitudinal study revealed a negative association between alexithymia and the 7.5-year incidence of compensated claims for low back pain, which was assessed by physician-confirmed diagnoses from administrative workers' compensation data. As the authors mentioned, a possible interpretation of their results is that alexithymic patients with chronic pain were unlikely to complain by filing a workers' compensated claim for low back pain injury because of their fear of being shamed and self-devaluated and/or their shyness and anxiety concerning the verbal expression of their emotions. Further prospective longitudinal studies with an appropriate method for estimating chronic pain are warranted.

### 2. Alexithymia, negative affect, and pain

Alexithymia is a personality trait associated with poor emotional awareness and affect regulation [9]. Our present findings confirm that this trait — in particular the aspect of alexithymia that involves having difficulty identifying one's feelings — is associated with the presence of chronic pain in the general population. This association also becomes nonsignificant when negative affect is controlled, suggesting that negative feelings such as depression and anxiety may mediate the association between alexithymia and chronic pain. This pattern of findings is consistent with previous research involving samples of individuals with chronic pain [13], [36].

In addition, our finding that the DIF and DDF domains of the TAS-20 were associated with the likelihood of chronic pain, while the EOT domain was not, is consistent with the findings of previous studies examining alexithymia and pain [7], [8], [11], [14]. The finding may be due to poor reliability of the EOT subscale [37]. Given the cross-cultural consistency of the finding, however, it does not appear to be due to language issues or cultural differences.

### 3. Possible mechanism underlying the association between alexithymia and chronic pain

Various theories linking alexithymia and physical illness have been conceptualized at the physiological level (e.g., the hypothalamic-pituitary-adrenal axis, chronic sympathetic hyperarousal, inflammation, and impaired immune status), the behavioral level, and the cognitive level (e.g., illness behavior, somatic amplification) [38], [39]. Some neuroimaging studies of alexithymia and chronic pain have been conducted recently, and their findings may contribute to our understanding of the mechanism of the relationship. First, neuroimaging data indicate not only hyperactivity in pain perception areas such as the insular cortex, but also hypoactivity in pain-processing regulatory areas such as the prefrontal cortex. Lack of an emotional regulation system might cause hypersensitivity to aversive bodily sensations and prolonged, pain-related affective reactions such as distress [40], [41]. Second, a possibility is related to the known negative effect of depression on the descending inhibitory system [42]. That is, alexithymia may lead to increased risk of depression, which may then interfere with an individual's ability to reduce or inhibit pain.

### 4. Clinical implications

Our analyses of the subgroup of participants with chronic pain supported a link between alexithymia and a number of measures of the key functioning domains in these individuals, including pain intensity, disability, depression and anxiety (positive associations), and life satisfaction (negative association). To the extent that these associations are causal — a conclusion that cannot yet be drawn due to the correlational nature of the current and previous findings — then treatments that decrease alexithymia could potentially have significant benefits across multiple quality of life domains for individuals with chronic pain. Thus, our findings support the need for research to develop and test interventions [43], [44] that could help individuals identify and describe their feelings, and to determine whether these interventions promote health-related quality of life and reduce the risk for chronic pain as a general health policy.

### 5. Study strengths and limitations

The study has a number of important strengths, including its large sample size and a population-based study design. Some limitations should be noted, however.

One primary limitation is that the data are cross-sectional. We thus cannot conclusively determine if alexithymia influences the presence and severity of negative affect and pain, if negative affect and pain influence alexithymia, or if there is an unidentified third variable that influences all three. However, experts generally agree that alexithymia is a trait that develops early in life and that it rarely changes without active intervention [45]. Thus, the possibility that alexithymia has a greater impact on pain and depression than these variables have on alexithymia remains viable. Prospective longitudinal studies are needed to clarify the contribution of alexithymia to the development of chronic pain and other negative outcomes. A second limitation is related to the possibility of selection bias, because approximately one-half of the individuals who participated in the regular Hisayama Study survey did not participate in our research. Certainly, we cannot deny the possibility that people with physical or mental complaints were more willing to participate in the study than were people without. In contrast, health-conscious people might have been more likely to participate in the study than non-health-conscious people. The fact that the present study population had many more females than males (601 women, 326 men) may support these possibilities [46]. Therefore, the generalizability of our findings to all individuals in the community may be limited. Nevertheless, we believe that our findings provide important information to consider alexithymia as a cognitive factor that may exacerbate physical symptoms such as chronic pain. Third, our questions about the presence of chronic pain have not clearly determined temporal patterns of chronic pain (e.g., it is unclear how a patient with recurrent pain would respond to the questions). A fourth limitation is that the causes of pain were not assessed in this study. It will be informative to explore whether or not the magnitude of the associations between alexithymia and chronic pain is different between participants with pain disorders that have or do not have one or more established biological causes. However, this limitation is unlikely to alter our conclusion, because previous studies have shown a positive correlation between alexithymia and pain-related outcomes regardless of the presence or absence of biological cause [12], [26], [27], [44], [47]. Lastly, pain intensity, disability, and life satisfaction were each assessed with a single-item measure using a VAS, which may have limited reliability of a part of the results compared to assessment that uses multiple-item questionnaires.

## Conclusions

The results of the present study indicate that alexithymia is significantly associated with a greater prevalence of chronic pain in the general population and that individuals with alexithymia have more pain intensity, disability, and depression and anxiety symptoms, and less life satisfaction than those without alexithymia. Our findings highlight certain clinically important concepts; i.e., that adverse psychological factors and personality traits play a significant role in the etiology of chronic pain. The early identification of alexithymia and negative affect may be beneficial in preventing chronic pain and reducing the clinical and economic burdens of chronic pain. Further prospective studies and interventional studies are needed to confirm this hypothesis.

## Supporting Information

Figure S1 **Flow chart of the participant recruitment.**
(EPS)
